# Antibacterial Ferroelectric Hybrid Membranes Fabricated via Electrospinning for Wound Healing

**DOI:** 10.3390/membranes11120986

**Published:** 2021-12-17

**Authors:** Ivan V. Lukiev, Ludmila S. Antipina, Semen I. Goreninskii, Tamara S. Tverdokhlebova, Dmitry V. Vasilchenko, Anna L. Nemoykina, Daria A. Goncharova, Valery A. Svetlichnyi, Georgiy T. Dambaev, Vyacheslav M. Bouznik, Evgeny N. Bolbasov

**Affiliations:** 1 B.P. Veinberg Research and Educational Centre, Tomsk Polytechnic University, 634050 Tomsk, Russia; ivl8@tpu.ru (I.V.L.); sig1@tpu.ru (S.I.G.); tst3@tpu.ru (T.S.T.); 2 Center for Chemical Engineering, ITMO University, 197101 St. Petersburg, Russia; 3 Department of Hospital Surgery with the Course of Cardiovascular Surgery, Siberian State Medical University, 634050 Tomsk, Russia; Ant_sv@mail.ru (L.S.A.); ahhoo14@mail.ru (D.V.V.); dambaev@vtomske.ru (G.T.D.); 4 N.M. Kizhner Research and Educational Centre, Tomsk Polytechnic University, 634050 Tomsk, Russia; 5 Laboratory of Biopolymers and Biotechnology, Chemical Faculty, Tomsk State University, 634050 Tomsk, Russia; nemoykina@rambler.ru; 6 Laboratory of Advanced Materials and Technology, Siberian Physical-Technical Institute, Tomsk State University, 634050 Tomsk, Russia; dg_va@list.ru (D.A.G.); v_svetlichnyi@bk.ru (V.A.S.); 7 Arctic Climate Materials Division, All Russian Scientific Research Institute of Aviation Materials, 105005 Moscow, Russia; bouznik@ngs.ru; 8 Department of Inorganic Chemistry, Tomsk State University, 634050 Tomsk, Russia; 9 Microwave Photonics Laboratory, V.E. Zuev Institute of Atmospheric Optics SB RAS, 634055 Tomsk, Russia

**Keywords:** ferroelectrics, electrospinning, nanofibers, wound healing

## Abstract

In the present study, wound healing ferroelectric membranes doped with zinc oxide nanoparticles were fabricated from vinylidene fluoride-tetrafluoroethylene copolymer and polyvinylpyrrolidone using the electrospinning technique. Five different ratios of vinylidene fluoride-tetrafluoroethylene to polyvinylpyrrolidone were used to control the properties of the membranes at a constant zinc oxide nanoparticle content. It was found that an increase of polyvinylpyrrolidone content leads to a decrease of the spinning solution conductivity and viscosity, causing a decrease of the average fiber diameter and reducing their strength and elongation. By means of X-ray diffraction and infrared spectroscopy, it was revealed that increased polyvinylpyrrolidone content leads to difficulty in crystallization of the vinylidene fluoride-tetrafluoroethylene copolymer in the ferroelectric β-phase in membranes. Changing the ratio of vinylidene fluoride-tetrafluoroethylene copolymer and polyvinylpyrrolidone with a constant content of zinc oxide nanoparticles is an effective approach to control the antibacterial properties of membranes towards *Staphylococcus aureus*. After carrying out in vivo experiments, we found that ferroelectric hybrid membranes, containing from five to ten mass percent of PVP, have the greatest wound-healing effect for the healing of purulent wounds.

## 1. Introduction

Human skin is the organ with the highest area acting as a natural barrier, which protects inner organs and tissues from various (physical, chemical, and biological) environmental factors. Due to its protective function, the skin often undergoes injury and as a consequence untreated or incorrectly treated wounds may result in developing significant local or systemic diseases [1]. The problem is extremely relevant for older and diabetic patients with chronic wounds [2].

Nowadays, the strategy of skin injury treatment is based on the prevention or elimination of the infection combined with an accelerated healing process for maximum structural and functional recovery [3]. Semi-permeable electrospun polymer membranes are of great interest as multifunctional materials for wound healing [4,5,6]. Compared to conventional dressings, these materials possess the following important characteristics: provide controllable release of antibacterial component, may be easily tailored to the wound form, provide long-term gas-exchange level and exudate sorption, and simulate the extracellular matrix structure thus enhancing wound regeneration [7]. With respect to that, pieso- and ferroelectric polymer membranes have gained a special interest of researchers during recent years [8,9]. Having the benefits of electrospun polymer membranes, such materials provide electrostimulation of the tissue healing process under mechanical, thermal, and electromagnetic stimuli and they do not require external power sources, thus preventing the accumulation of electrolysis products in the tissue [10,11].

Vinylidene fluoride-tetrafluoroethylene (VDF-TeFe) copolymer is one of the most electrically active polymers demonstrating high residual polarization and piezoelectric constants [12,13]. At the same time, VDF-TeFe copolymer demonstrates the lowest polarization switch period [14], high Curie temperature, high strength, thermal stability, tissue compatibility, and anti-adhesive properties [15,16]. The complex of these properties makes VDF-TeFe copolymer one of the most potentially useful materials for the development of electrospun polymer membranes with ferro- and piezoelectric properties for wound healing.

The disadvantage of VDF-TeFe copolymer semi-permeable membranes is in their extreme chemical inertness, low solubility, and the lack of ability to preserve their properties under treatment with concentrated and diluted acidic, basic and neutral electrolytes [17]. These factors hinder the loading of pharmacologically active antibacterial and regenerative agents. Fabrication of composite membranes based on VDF-TeFe and hydrophilic polymer, which provide encapsulation and effective delivery of antibacterial and regenerative components in the injury site, may be a satisfactory solution to the problem.

Polyvinylpyrrolidone (PVP), which is a synthetic polymer comprising of 1-vinyl-2-pyrrolidon monomers, may be utilized as hydrophilic polymer. PVP is a non-toxic biocompatible polymer with good solubility in water and various organic solvents, medium conductivity and charge transport ability, and substantive to complex hydrophilic and hydrophobic compounds [18]. These properties determine the wide applicability of PVP in the development of biomaterials and pharmaceutical formulations [19].

ZnO nanoparticles may be used as an antibacterial agent for enhanced wound epithelization. This material possesses high antibacterial properties [20], better stability during the storage and production process compared to antibiotics [21], with ferro- and piezoelectric properties [22], which make it suitable for the development of composite piezoelectric membranes for wound healing.

The aim of the present work was to reveal the possibility of fabrication of ZnO-loaded VDF-TeFe/PVP hybrid piezoelectric membranes with controllable antibacterial activity varied by the VDF-TeFe/PVP ratio in the VDF-TeFe/PVP/ZnO membrane. The effects of the polymer ratio on the structure, physico-chemical properties, antibacterial properties, and the ability of the membrane to restore skin tissue in the case of abundant contamination were also studied.

## 2. Materials and Methods

### 2.1. Membrane Fabrication

First, the solvent mixture for the preparation of the spinning solution was prepared. To do that, acetone (EKOS-1, Moscow, Russia) and isopropanol (EKOS-1, Moscow, Russia) were mixed in mass ratio 80/20 using a magnetic stirrer (EKOS-1, Moscow, Russia). The solvents were mixed at room temperature for 4 h. Then, the suspension of ZnO nanoparticles in dimethylformamide (DMFA, EKOS-1, Moscow, Russia) was prepared. 0.45 ± 0.01 g of ZnO nanoparticle powder was placed in a 200 mL glass hermetically sealed reactor and 3.0 ± 0.1 g of DMFA were added. The reactor was placed in an ultrasonic bath (Sapphire 5M, Saint-Petersburg, Russia) and ultrasonicated for 12 h at a temperature of 50 °C. Then the reactor was cooled to room temperature. Next, 47.0 ± 0.1 g of the solvent mixture and 2.55 ± 0.01 g of the polymers were placed in the reactor, which was further sealed and subjected to another 12 h of ultrasonication at a temperature of 50 °C.

ZnO nanopowder (average nanoparticle size of 18–26 nm) was obtained using zinc target laser ablation in an air atmosphere as reported previously [23]. Vinylidene fluoride-tetrafluoroethylene (VDF-TeFe) copolymer (GaloPolymer, Moscow, Russia) and polyvinylpyrrolidone (PVP) (Kollidon^®^ 17 PF, BASF, Ludwigshafen am Rhein, Germany) were used as polymer components. Five types of the spinning solutions (with PVP content in VDF-TeFE/PVP composites of 0, 5, 10, 20, and 40 wt% respectively) were prepared for the experiments. An SV-10 viscometer (AND, Tokyo, Japan) was used for the measurements of the spinning solution viscosity. An InoLab Cond 7319 conductometer with a TetraCon 325 measuring cell (WTW, Weilheim, Germany) was used for the conductivity measurements. Viscosity and conductivity of the spinning solutions were measured at 24 °C.

The membranes were produced using a NANON-01A electrospinning setup (MECC Co., Ltd., Fukuoka, Japan). An aluminum cylinder with a diameter of 200 mm and a length of 100 mm was used to collect nanofibers. The following parameters were used for the membrane fabrication: applied voltage of 30 kV, injector-to-collector distance of 40 mm, spinning solution feed rate of 4 mL/h, collector rotation rate of 200 rpm. A 22 G needle was used as injector. The electrospun membranes were exposed to vacuum at a pressure of 10^−2^ Pa and 100 °C for 10 h to remove the residual solvent.

### 2.2. Physico-Chemical Characterization

#### 2.2.1. Scanning Electron Microscopy (SEM)

The sample morphology was studied using a JCM-6000 (JEOL, Tokyo, Japan) electron microscope. Before microscopy, the samples were coated with thin gold layer in an SC7640 magnetron sputtering system (Quorum Technologies Ltd., Laughton, UK). The average fiber diameter was calculated from the captured SEM images using ImageJ 1.38 software (National Institutes of Health, Bethesda, MD, USA) from not less than 400 measurements.

#### 2.2.2. Energy-Dispersive Spectroscopy (EDS)

Energy-dispersive spectroscopy (EDS) (JED 2300, JEOL, Tokyo, Japan) was used for the analysis of the chemical composition of the fabricated samples.

#### 2.2.3. Surface Wetting

The water contact angle of the fabricated materials was measured using an EasyDrop-100 (Krüss GmbH, Hamburg, Germany) optical goniometer. The measurement was performed 1 min after the 3 μL drop of Milli-Q water had been placed on the sample surface.

#### 2.2.4. Tensile Testing

Tensile testing of the fabricated membranes was conducted according to ISO 9073-3:1989 “Textiles—Test methods for nonwovens—Part 3: Determination of tensile strength and elongation” using an Instron 3344 (Instron, Buckinghamshire, UK) testing machine with a 0.10 ± 0.01 N sample pre-load.

#### 2.2.5. Fourier-Transform Infrared Spectroscopy (FTIR)

Chemical structure of the fabricated materials was studied using a Tensor 27 (Bruker, Ettlingen, Germany) FTIR system equipped with PIKE MIRacle (Bruker, Ettlingen, Germany) ZnSe crystal ATR accessory. The spectra were recorded in a range of 600–1800 cm^−1^ with a resolution of 2 cm^−1^ and treated using OPUS 3D (Bruker, Ettlingen, Germany) software.

#### 2.2.6. X-ray Diffraction Analysis (XRD)

The crystal structure of the composite membranes was investigated using XRD 6000 (Shimadzu, Kyoto, Japan) equipment with a Cu Kα (1.54056 Å) radiation source. The average crystallite size was calculated from the Debye–Scherrer Equation (1):

(1) $$l_{c}=\frac{k\lambda }{\cos\theta \sqrt{\beta^{2}-\beta_{r}^{2}}}$$

where *λ* is the X-ray wavelength (Cu K-alpha, λ = 1.54056 Å), *β* is the line broadening at half the maximum intensity, *β_r_* is the broadening reflex of the apparatus = 0.1, *θ* is the Bragg angle, and *k* = 0.9.

### 2.3. Biomedical Studies

#### 2.3.1. Antibacterial Activity

Antibacterial activity of the fabricated materials was studied according to ISO 20743:2013 “Textiles—Determination of antibacterial activity of textile products”. *Staphylococcus aureus* (ATCC 25923) was cultured with 2 × 2 cm^2^ samples as described previously [23]. Antibacterial activity A was measured using Equation (2):

(2) $$A=(\mathrm{lg}C_{t}-\mathrm{lg}C_{0})-(\mathrm{lg}T_{t}-\mathrm{lg}T_{0})=F-G$$

where F = (lgC_t_ − lgC_0_) is the growth rate on the control (PVP-free sample); lgC_t_ is the average decimal logarithm of the number of bacteria found on the three control samples incubated for 24 h; lgC_0_ is the average decimal logarithm of the number of bacteria observed on the three control samples immediately upon seeding with bacteria; G = (lgT_t_ − lgT_0_) the growth rate on the VDF-TeFE/PVP/ZnO; lgT_t_ the average value of the decimal logarithm of the number of bacteria observed after incubation for 24 h on the three treated samples; and lgT_0_ the average decimal logarithm of the bacteria number observed immediately after bacteria seeding on the three VDF-TeFE/PVP/ZnO samples. The general assessment criteria follow a definition by the Hohenstein Institutes, in that a growth reduction efficacy of <0.5 corresponds to no antibacterial activity, whereas ≥0.5 to <1 corresponds to slight, ≥1 to <3 to significant, and a growth reduction of ≥3 indicates a strong antibacterial activity, respectively [24].

#### 2.3.2. In Vivo Contaminated Full-Thickness Wound Healing

The wound healing activity of the fabricated materials was studied on 30 adult 180–200 g Wistar rats. The rats were anesthetized and a rectangular excision area with size of 20 × 20 mm^2^ was cut on each animal. The edges of the wounds and underlying muscles were crushed with Kocher’s forceps. After that, a microbial suspension containing 10^6^ colony-forming units (CFU) of *Staphylococcus aureus* was applied topically to the wound area. The surface of the wound was covered with a plastic wrap for 72 h to form an acute inflammation. Animals were divided into 6 groups with 5 animals in each group. For the animals of the control group, a gauze bandage soaked in an aqueous solution of chlorhexidine (Kemerovo pharmaceutical factory, Kemerovo, Russia) was applied to the wound surface. The dressings were changed on days 3, 5, and 10 of the experiment. Images of the wound under the membrane were obtained using a digital camera EOS 250D (Canon, Tokyo, Japan). The ImageJ program was used to estimate the area of the wound under the membrane. The study was carried out in accordance with the principles of humane treatment of laboratory animals described in [25]. Prior to investigation, all membrane samples were sterilized in an ethylene oxide atmosphere using a gas sterilizer AN4000 (Andersen Products Ltd., Clacton-on-Sea, UK).

### 2.4. Statistical Analysis

The data were analyzed with Prism 7 (GraphPad software, San Diego, CA, USA) using one-way ANOVA with Tukey’s correction for multiple comparisons. Differences were considered significant at *p* < 0.05.

## 3. Results and Discussion

**Figure 1:**
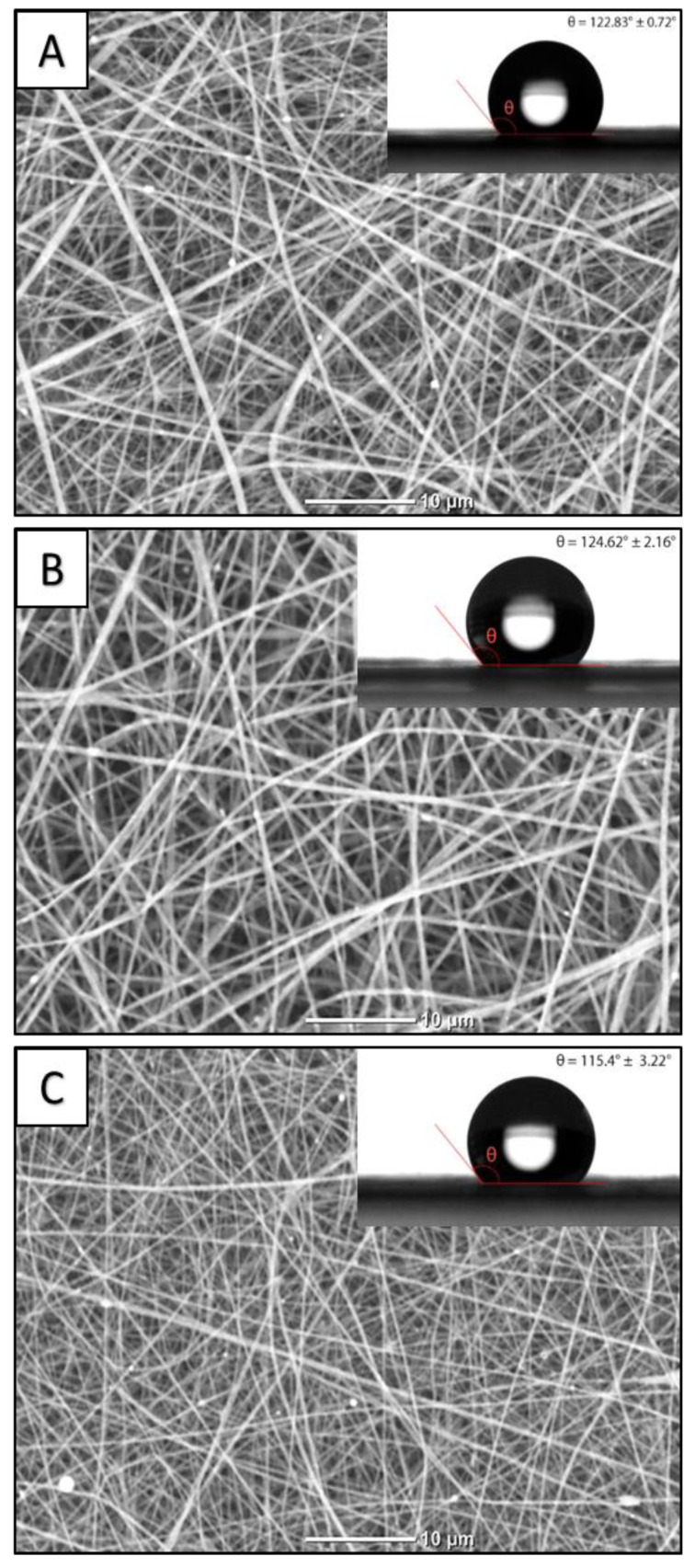
SEM images and water contact angles of VDF-TeFE/ZnO membranes with various PVP content: (**A**) 0 wt%; (**B**) 10 wt%; (**C**) 40 wt%.

SEM images of the fabricated VDF-TeFE/ZnO membranes with various VDF-TeFE ratios are shown in Figure 1. The increase of PVP content in the spinning solution up to 5 wt% results in ≈14% increase of the spinning solution dynamic viscosity compared to control (Table 1). These changes may be explained by possible intermolecular interactions between the macromolecules and solvents. Further increase of PVP content leads to a reduced dynamic viscosity, which is a result of the high-molecular VDF-TeFE component. With that, the rise of PVP content results in the reduction of the spinning solution conductivity. Regardless of the PVP content in the spinning solution, all membranes were formed by cylindrical fibers of regular shape, randomly intertwining with each other. The highest average fiber diameter was observed for the membranes containing 5 wt% of PVP fabricated from the most viscous spinning solution. VDF-TeFE/PVP/ZnO membrane, which was fabricated from the solution containing 40 wt% of PVP having the lowest conductivity and viscosity demonstrating the lowest average fiber diameter (Table 1). Taking into account the fact that the conductivity of the spinning solutions containing 40 wt% of PVP is ≈20% less compared to PVP-free solution (while their viscosity is 8-fold less), it may be concluded that under selected fabrication parameters the fiber diameter of VDF-TeFE/PVP/ZnO membranes is mainly affected by the spinning solution viscosity.

The conducted studies demonstrate that the maximum tensile strength and elongation were observed by PVP-free membranes from the control group. With the increase of PVP content up to 40 wt% these characteristics were reduced for ≈50% compared to the control group. These changes may be explained by the formation of defects and the disordered crystal structure of VDF-TeFE copolymer in the fiber.

Elemental composition of the fabricated VDF-TeFE/PVP/ZnO membranes is presented in Table 2. The elemental composition of the control membrane is presented by carbon and fluorine, the main elements, which form the VDF-TeFE macromolecule. Oxygen and zinc are the elements of the ZnO inorganic additive. The increase of PVP content is accompanied by the appearance of nitrogen in the elemental composition of the fabricated membranes.

Moreover, this results in the rise of oxygen content followed by the reduction of F/O and F/C ratios, which demonstrates the fabrication of the composite membranes. With that, regardless of PVP content, the Zn concentration does not vary significantly (Table 2). The obtained results confirm the fabrication of VDF-TeFE/PVP/ZnO composite membranes with equal ZnO concentration.

**Figure 2:**
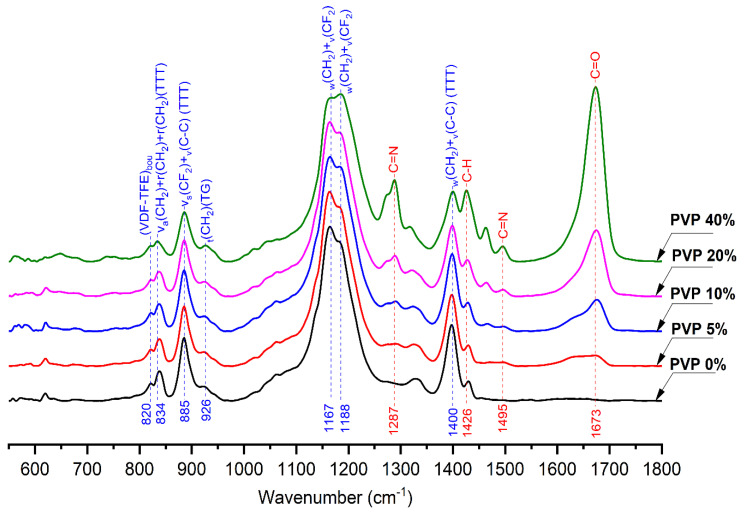
IR spectra of VDF-TeFE/PVP/ZnO membranes with various PVP content.

Infrared spectroscopy allows the determination of the chemical composition of the polymer membranes as well as finding the VDF-TeFE macromolecule conformations responsible for the ferroelectric phase formation [26]. Infrared spectra of the fabricated membranes are shown in Figure 2. In the spectrum of the control VDF-TeFE membrane two intensive bands at 1165 cm^−1^ and 1188 cm^−1^_,_ which correspond to the superposition of twisting and stretching vibrations in CH_2_ and CF_2_ bonds, were observed. An intensive band at 1398 cm^−1^ refers to the superposition of wagging and asymmetric stretching vibrations in CH_2_ and C–C groups. The band at 884 cm^−1^ corresponds to the superposition of antisymmetric stretching and ricking vibrations in the CH_2_ group. The low-intensity band at 840 cm^−1^ refers to the superposition of symmetric stretching in CF_2_ and C–C groups [27,28]. The presence of the intensive bands at 840, 884, and 1398 cm^−1^ corresponding to *trans*-conformation and the low-intensity band at 925 cm^−1^ corresponding to *gauche*-conformation confirm the flat zigzag conformation of the VDF-TeFE macromolecule with a high dipole moment perpendicular to the macromolecule axis [29].

With the increase of PVP content, the bands corresponding to PVP occur: 1650 cm^−1^ stretching vibration of the C=O in the pyrrolidone group, 1421 cm^−1^ and 1372 cm^−1^ correspond to the CH deformation modes from the CH_2_ group in PVP, 1287 cm^−1^ which is related to the C–N bending vibration from the pyrrolidone structure, the band at about 1495 cm^−1^ refers to the characteristic vibration of C=N (pyridine ring) [30,31]. The presence of bands at 840, 884, and 1398 cm^−1^ and the absence of shifts regardless of PVP content is evidence for the preservation of the *trans*-conformation of VDF-TeFE molecules with high dipole moment. The presence of the bands corresponding both to PVP and VDF-TeFE confirms the fabrication of composite membranes as well as the results of EDX studies. It should be noted that the addition of hydrophilic PVP in the range of 0 to 40 wt% had no effect on the surface properties of the fabricated membranes, which is demonstrated by the insignificant changes in water contact angles (122° for the control membrane and 110° for the membranes containing 40 wt% of PVP) (Figure 1).

**Figure 3:**
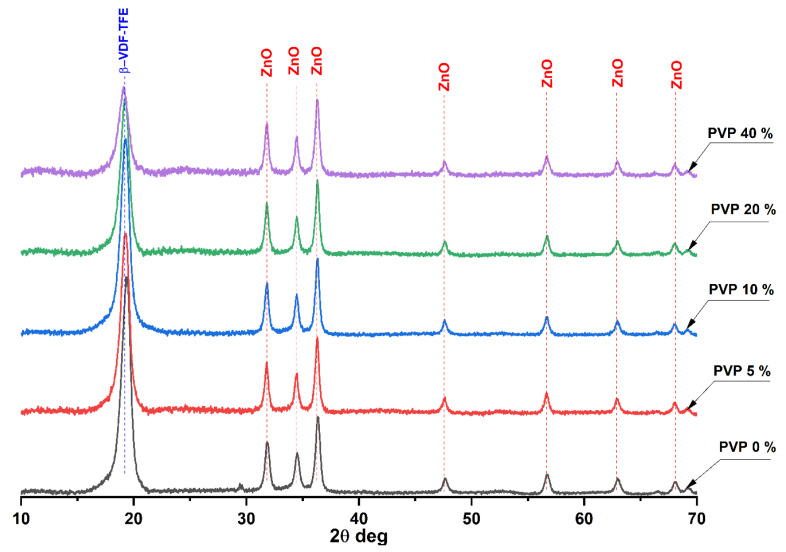
XRD patterns of the VDF-TeFE/PVP/ZnO membranes with various PVP contents.

The XRD patterns of the fabricated membranes are presented in Figure 3.

On the diffractogram of the PVP-free membrane several intensive reflexes were observed. The most intensive one at 19.3° corresponds to the most electrically active ferroelectric β-phase of the VDF-TeFE copolymer formed by macromolecules in the *TTT* conformation. The reflexes at 31.7°, 34.4°, 36.3°, 47.6°, 56.7°, 62.9°, and 68.0° correspond to wurtzite, crystalline ZnO formed during the laser ablation of the zinc target in air [32]. The formation of the VDF-TeFE β-phase in the membrane fibers is due to polarization of VDF-TeFE macromolecules under the high voltage field in the space between the needle and collector, as well as the high tension stress and solvent evaporation rate during the electrospinning process [33].

The increase of PVP concentration in the fabricated membranes results in widening of the VDF-TeFE β-phase reflex, which is due to the reduction of crystallite size by more than 30% in the membranes containing 40 wt% of PVP compared to control (Table 3).

Thus, the rise of PVP content hinders VDF-TeFE crystallization to the ferroelectric β-phase, which is due to the formation of interlayers because of intermolecular interactions between the polymers in the fiber. However, even at PVP concentration of 40 wt%, VDF-TeFE/PVP/ZnO membranes have a ferroelectric crystal structure, which is evidenced by the absence of shifts of β-phase reflex (Figure 3). As it was expected, the ZnO nanoparticle crystal structure is not affected by the PVP concentration, which is obvious from the constant position of its reflexes and crystallite size (Figure 3, Table 3).

The results of antibacterial activity studies of the fabricated membranes toward Gram-positive *S. aureus* pathogen are presented in Table 4. The membranes from the control group and the membranes containing 5 wt% of PVP demonstrated low antibacterial activity. The increase of PVP concentration from 10 to 40 wt% changed the activity of the membranes from bacteriostatic to bactericidal (Table 4). The observed variations of antibacterial activity are due to the following factors. It is well known that antibacterial action of ZnO nanoparticles is based on cell wall misfunction and destruction because of Zn^2+^ ions release, electrostatic accumulation of the particles, and reactive oxygen species (ROS) formation, which penetrate inside the cell inducing its death. With that, nanoparticles internalization is induced with the increase of antibacterial agent concentration and the decrease of its size [34]. PVP is a water-soluble hydrophilic polymer, which is released to the medium [35] thus providing a transport of ZnO nanoparticles to the contamination zone. In such a manner, the increase of PVP content improves ZnO nanoparticle transport to the contamination zone and increases their concentration, thus enhancing the antibacterial activity of the fabricated membranes. The second factor is possible “lysosomal storage disease” due to the PVP accumulation in the cells, which inhibits cell vital functions inducing their death [36]. The third factor is possible formation of complexes between the protonated PVP with biologically active anions [37] reducing their availability for cells.

**Figure 4:**
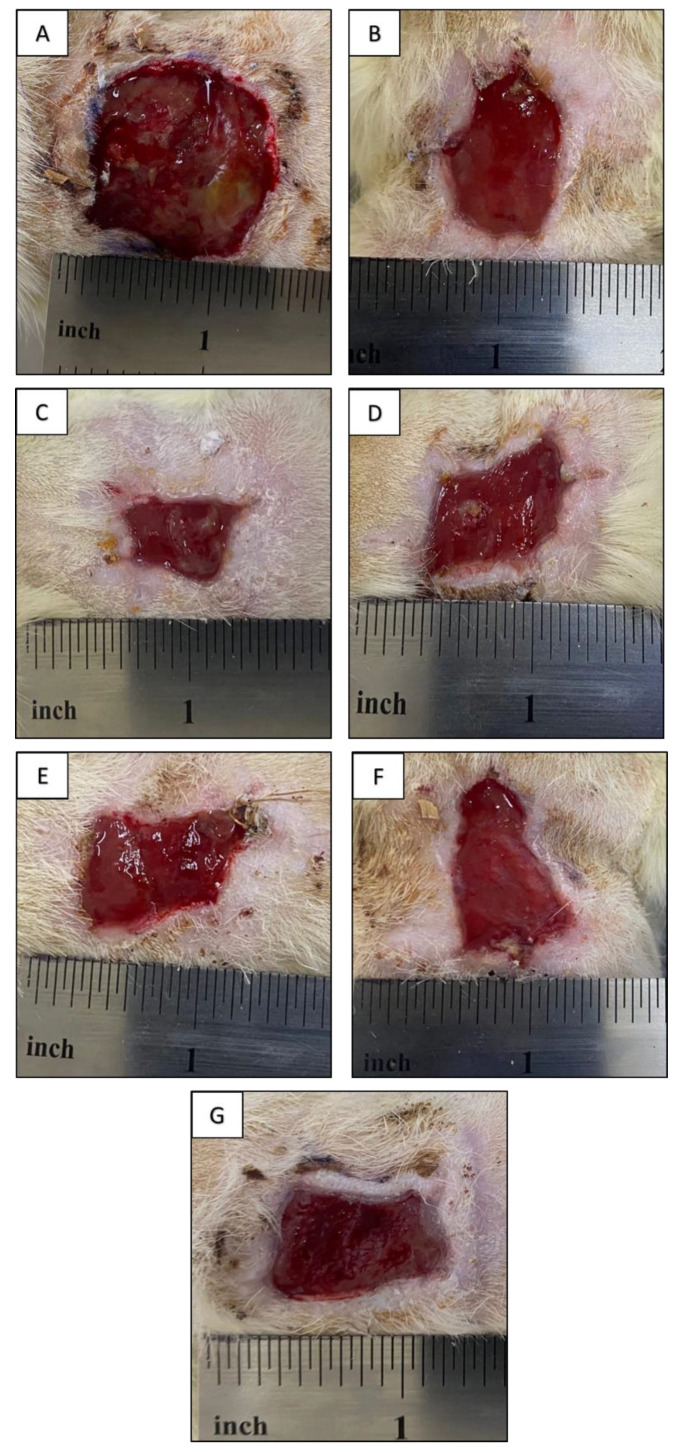
Photographs of the wounds after 10 days of the experiment: (**A**)—intact wound, (**B**)—after contact with the material containing 0 wt% of PVP, (**C**)—5 wt% of PVP, (**D**)—10 wt% of PVP, (**E**)—20 wt% of PVP, (**F**)—40 wt% of PVP, (**G**)—control group.

The photographs of the original wound as well as the wounds after 10 days of contact with VDF-TeFE/PVP/ZnO membrane bondages and chlorohexidine (control group) are presented in Figure 4. The formed septic wound appears as a purulo-necrotic acute inflammation site, which is evidenced by gray-green incrustations and the bad odor of the wound surface. The wound area was found to be at 85 ± 12 mm^2^ (Figure 4A). After 10 days of the experiment, the bed of the wound contacted with PVP-free membrane was found to be clear, membrane removal was not accompanied by wound traumatization. Active epithelization of the wound periphery with moderate granular tissue formation was observed. The wound area was found at 44 ± 8 mm^2^.

After 10 days of contact with VDF-TeFE/PVP/ZnO containing 5 wt% of PVP, the wound area was found at 24 ± 4 mm^2^ (Figure 4C). The wound bed was clean, active wound periphery epithelization and moderate granulation were observed. The membrane was removed without wound traumatization. The area of the wound contacted with VDF-TeFE/PVP/ZnO bondage membrane containing 10 wt% of PVP was found at 31 ± 6 mm^2^. The wound bed was clean, granular tissue formation and epithelization were observed (Figure 4D). In the case of the membrane containing 20 wt% of PVP, a full-frap musculodermic wound with area of 42 ± 5 mm^2^ was formed. The membrane removal was accompanied by slight traumatization of the wound. Fibrin-containing zones were observed on the wound bed. Active epithelization was observed on the wound periphery (Figure 4E). After the application of VDF-TeFE/PVP/ZnO membrane containing 40 wt% of PVP, fibrin-coated areas of up to 2 mm^2^ were found. Active epithelization was observed on the wound periphery. The wound area was found at 46 ± 4 mm^2^ (Figure 4F). The wound treated with “classic” chlorohexidine-soaked gauze had an area of 38 ± 6 mm^2^. The bondage was removed with wound traumatization. The wound bed was clean. Active epithelization, granulation and low fibrin formation were observed on the wound periphery.

The conducted studies demonstrated better conditions for wound regeneration under VDF-TeFE/PVP/ZnO membranes compared to chlorohexidine-soaked gauze. With that, the membranes containing from 5 to 10 wt% of PVP exhibited the best regeneration ability. This fact may be explained by the ability of these membranes to preserve their structural integrity during the exposure to model media [38] as well as to provide necessary transport of ZnO nanoparticles to the contamination site for the suppression of pathological microbial flora. Combination of the abovementioned features allows these membranes to support optimal wound humidity and gas exchange with the environment, high exudate sorption ability, and low adhesion to the wound surface, thus providing optimal conditions for wound regeneration. It is known that piezoelectric PVDF-based membranes may exhibit negative effects on *Staphylococcus* under dynamic loads even without antibacterial agents [39]. As *trans* VDF-TeFE conformation prevails in the membranes containing from 5 to 10 wt% of PVP (Figure 2), and the crystal structure is mainly presented by ferroelectric crystallites (Figure 3) it may be suggested that the decrease of pathogen concentration and enhanced tissue regeneration is due to the piezoelectric properties of the fabricated membranes. It is also known that under external mechanical stimuli, piezoelectric scaffolds promote migration, adhesion, and cytokine secretion in human fibroblasts in vitro [40]. The ability of piezoelectric scaffolds to generate electrical impulses in response to the mechanical action on the tissue surrounding the implant allows promotion of wound healing regardless of the implantation zone [9]. Thus, optimal chemical and electrical properties of the fabricated membranes enable intensifying regeneration processes in the wound site.

The increase of PVP content from 20 to 40 wt% results in the impaired membranes long-term stability in model media [38]. This fact hinders the tissue regeneration process under such membranes. With that, enhanced adhesion of these membranes to the wound surface leads to wound traumatization hindering its regeneration. Moreover, it was revealed that high PVP content hinders VDF-TeFE crystallization into the ferroelectric β-phase, which should lower the piezoelectric properties of the membranes [41,42] as well as their healing potential. Thus, the conducted studies demonstrate that the optimal PVP content in VDF-TeFE/PVP/ZnO composite membranes for wound regeneration lies in the range of 5–10 wt%.

## 4. Conclusions

In the present study, wound healing ferroelectric membranes doped with zinc oxide nanoparticles were fabricated from vinylidene fluoride-tetrafluoroethylene copolymer and polyvinylpyrrolidone using the electrospinning technique. It has been shown that changing the ratio of electrically active vinylidene fluoride-tetrafluoroethylene copolymer and water-soluble polyvinylpyrrolidone (from 100/0 to 60/40) makes it possible to effectively vary the physicochemical and biomedical parameters of the obtained materials. Polyvinylpyrrolidone hindered the crystallization of vinylidene fluoride-tetrafluoroethylene copolymer, which led to a deterioration in the mechanical characteristics of the membranes (nearly two-fold decrease in tensile strength and elongation). The surface of the obtained materials had an extremely hydrophobic character (water contact angle ≈120°) regardless of the polyvinylpyrrolidone content. The polyvinylpyrrolidone addition made it possible to change the antibacterial activity of the formed materials against *Staphylococcus aureus* from bacteriostatic to bactericidal. In vivo studies using the rat model showed a positive effect of the formed membranes on the healing of wounds with abundant infection. It was shown that the optimal concentration of polyvinylpyrrolidone lies between five to ten mass percent based on the combination of physicochemical and biomedical characteristics.

## Figures and Tables

**Table 1:** Spinning solution viscosity and average fiber diameter, tensile strength, and elongation of the fabricated materials with various PVP contents.

PVP Content, %	Dynamic Viscosity, 10^−3^ Pa × s	Conductivity, µS/cm	Mean Fiber Diameter, µm	Tensile Strength, MPa	Elongation, %
0	51.9 ± 4.3	43.5 ± 1.0	0.36 ± 0.09	13.4 ± 0.8	70.0 ± 6.8
5	60.3 ± 2.5	38.2 ± 0.6	0.47 ± 0.11	10.9 ± 0.7	42.6 ± 4.7
10	52.8 ± 3.9	33.9 ± 0.7	0.41 ± 0.12	8.6 ± 1.1	59.9 ± 6.4
20	28.0 ± 1.5	32.8 ± 0.5	0.40 ± 0.08	9.2 ± 0.4	41.0 ± 3.4
40	6.3 ± 0.4	34.5 ± 0.5	0.32 ± 0.09	6.8 ± 0.7	36.8 ± 6.6

**Table 2:** Chemical composition of the fabricated membranes, studied by the EDX method, at%.

PVP Content, %	C	F	O	N	Zn	F/C	F/O
0	53.1 ± 1.5	41.3 ± 1.7	3.1 ± 0.2	-	2.6 ± 0.1	0.80 ± 0.05	13.56 ± 1.22
5	56.6 ± 2.5	36.4 ± 2.8	3.7 ± 0.1	0.7 ± 0.2	2.6 ± 0.1	0.64 ± 0.08	9.84 ± 0.98
10	59.9 ± 2.1	33.0 ± 2.3	4.1 ± 0.1	1.4 ± 0.1	2.5 ± 0.1	0.56 ± 0.06	8.10 ± 0.61
20	60.3 ± 0.4	29.1 ± 0.6	5.3 ± 0.2	2.7 ± 0.1	2.5 ± 0.1	0.48 ± 0.01	5.50 ± 0.29
40	66.6 ± 1.8	17.8 ± 1.9	7.4 ± 0.1	5.7 ± 0.2	2.5 ± 0.1	0.27 ± 0.04	2.42 ± 0.27

**Table 3:** Crystallite size in VDF-TeFE/PVP/ZnO membranes with various PVP contents.

PVP Content, %	Crystal Size, nm	
	β-Phase VDF-TeFE	ZnO
0	10.5 ± 1.2	20.2 ± 1.7
5	9.9 ± 0.9	20.9 ± 1.7
10	9.1 ± 0.7	20.9 ± 2.0
20	8.4± 1.1	20.4 ± 2.1
40	7.2 ± 1.5	20.5 ± 1.6

**Table 4:** Antibacterial activity of VDF-TeFE/PVP/ZnO membranes with various PVP contents.

Sample	Incubation Time, h	Number of Microorganisms, CFU/mL	Growth Rate on a Control Sample	Growth Rate on Hybrid Samples	Antibacterial Activity
PVP 0%	0	1.67 × 10^4^ ± 0.15	3.071	2.74	0.33
	24	2.00 × 10^7^ ± 0.50			
PVP 5%	0	1.40 × 10^4^ ± 0.10		2.82	0.25
	24	9.17 × 10^6^ ± 0.29			
PVP 10%	0	1.43 × 10^4^ ± 0.40		2.04	1.03
	24	1.53 × 10^6^ ± 0.06			
PVP 20%	0	1.60 × 10^4^ ± 0.17		1.09	1.98
	24	1.97 × 10^5^ ± 0.06			
PVP 40%	0	1.67 × 10^4^ ± 0.29		0.48	2.60
	24	5.00 × 10^4^ ± 1.00			

## Data Availability

Not applicable.
